# Stress hormone level and the welfare of captive European bison (*Bison bonasus*): the effects of visitor pressure and the social structure of herds

**DOI:** 10.1186/s13028-021-00589-9

**Published:** 2021-06-10

**Authors:** Daniel Klich, Rafał Łopucki, Marta Gałązka, Agnieszka Ścibior, Dorota Gołębiowska, Rita Brzezińska, Bartosz Kruszewski, Tadeusz Kaleta, Wanda Olech

**Affiliations:** 1 Department of Animal Genetics and Conservation, Institute of Animal Sciences, Warsaw University of Life Sciences, Ciszewskiego 8, 02-786 Warsaw, Poland; 2 Centre for Interdisciplinary Research, The John Paul II Catholic University of Lublin, Konstantynów 1J, 20-708 Lublin, Poland; 3 Department of Food Hygiene and Public Health Protection, Institute of Veterinary Medicine, Warsaw University of Life Sciences, Nowoursynowska 159, 02-776 Warsaw, Poland; 4 Warsaw Zoological Garden, Ratuszowa 1/3, 03-461 Warsaw, Poland; 5 Laboratory of Oxidative Stress, Centre for Interdisciplinary Research, The John Paul II Catholic University of Lublin, Konstantynów 1J, 20-708 Lublin, Poland; 6 Department of Chemistry, Institute of Food Sciences, Warsaw University of Life Sciences (SGGW), Nowoursynowska 159 C, 02-776 Warsaw, Poland; 7 Department of Food Technology and Assessment, Institute of Food Sciences, Warsaw University of Life Sciences (SGGW), Nowoursynowska 159 C, 02-776 Warsaw, Poland

**Keywords:** Aggression, Cortisol, Enclosure, Wisent, Parasitological infestation, Zoo, Feces

## Abstract

**Background:**

Captive European bison (*Bison bonasus*) play an active role in conservation measures for this species; this includes education, which may conflict with these animals’ welfare. The effect of the presence of visitors on the welfare of captive animals can be negative, positive or neutral. However, the response of a given species to visitors is difficult to predict, since even closely related species display varying levels of tolerance to captivity. The aim of the study was to compare immunoreactive fecal cortisol levels (regarded as an indicator of the level of physiological stress) in groups of captive European bison that differed in terms of their social structure and the level of visitor pressure. The second aim was to determine if there was a correlation between intestinal parasitic burden and immunoreactive fecal cortisol levels.

**Results:**

Immunoreactive fecal cortisol levels were not influenced by sex or age. However, study site and the interaction between study site and visitor pressure were statistically significant. European bison in one enclosure presented higher levels of immunoreactive fecal cortisol on weekdays than at weekends. In the other two study sites, the levels did not differ between weekdays and weekends. No correlation was found between parasitological infestation and immunoreactive fecal cortisol levels.

**Conclusions:**

Measurement of fecal cortisol metabolites could be a valuable method for further research into the welfare of European bison in captivity. More subtle factors such as individual animal characteristics, feeding systems, and the arrangement of enclosures can be of great importance in terms of the effect of visitors on animals. The results of this study can be used in guidelines for the management of European bison populations.

**Supplementary Information:**

The online version contains supplementary material available at 10.1186/s13028-021-00589-9.

## Background

Breeding of captive European bison (*Bison bonasus*) saved this species, which was extinct in the wild in 1919 [1]. As a result of conservation efforts, the wild population of European bison has spread across a large area of Europe [2, 3]. However, a significant proportion of the entire European bison population is made up of animals that are kept in captivity, therefore protecting these animals is also an important element of conservation strategies [4]. For this reason, methods of European bison breeding are constantly the subject of scientific considerations [5, 6]. Captive breeding allows individual mating to be planned, initial herds to be created, wild populations to be enriched with individuals of known origin (e.g., to increase the genetic diversity of free-ranging herds), and research to be conducted on the health of European bison [7–10]. Keeping animals in enclosures allows them to be presented to the public, which in turn may contribute to increased acceptance of the occurrence of wild herds in natural environments [11]. This educational role is realized by zoos and breeding farms, where European bison are presented in a quasi-natural environment. These centers are popular tourist attractions because the European bison is the largest mammal species in Europe and is deeply embedded in culture and art [12, 13].

The educational aspects of presenting captive European bison may, however, conflict with its welfare due to the almost permanent presence of visitors. The effect of visitors on the stress and welfare of exhibited animals has been shown for many captive species [14, 15]. However, the response of a given species to visitors is difficult to predict, since even closely related species show varying levels of tolerance to captivity [16]. Theoretically, the effect of the presence of visitors on captive animals’ welfare could be negative (e.g., stress-inducing), positive (as a kind of enrichment) or neutral; however, descriptions of negative effects dominate in the literature. Morgan and Tromborg [17] and Sade [18] noted that there are several sources of stress that are typical of captive animals, and a cumulative effect of stressors related to visitors can occur, e.g., the cumulative effect of the number of visitors, their behavior, the closeness of interaction with animals, and the noise produced. Detailed studies on the relations between visitors and animals kept in enclosures have mainly concerned primates and have revealed increased frequency of abnormal behavior, increased cortisol levels, or altered behavior as a result of visitor pressure [15, 19]. A negative reaction to visitor pressure was also often the case with captive ungulates, including social species [20–23]. On the other hand, observation of 12 species of ruminants in captivity showed only a slight reaction to visitors, but for some species this reaction depended on the sex of the animals [24].

The positive role of visitors in terms of their function as enrichment for captive animals is still being discussed in behavioral research [14, 15]. There are several species whose high level of curiosity leads them to approach visitors frequently. A particularly interesting case is the black-tailed prairie dog (*Cynomys ludvicianus*), which displays stronger positive reactions (adults kiss more and fight each other less) when there is a greater number of visitors [25]. Wood [26] observed positive changes in behavior in a group of captive chimpanzees (*Pan troglodytes*), and Owen [27] observed that play behavior was stimulated by visitors in the Asian short-clawed otter (*Aonyx cinerea*).

On the basis of the present research, it is difficult to predict the physiological response of European bison to the pressure of visitors and to find the right compromise between educational goals and the welfare of this species in captivity. It should be remembered that European bison naturally live in groups, therefore social relations are an important factor that affects the broadly understood welfare of this species [28]. In wild herds of European bison, an adult female has the role of group leader; however, some studies indicate collective decision-making [29], whereas aggression is usually associated with males [30, 31]. These behavioral features are usually taken into consideration when evaluating breeding strategies, and the general rule in captive herds is to keep one adult male in a family group to minimize aggression, especially between males. When these rules are not complied with, one extreme effect of adult male aggression is the killing of a young individual in the enclosure. For this reason, subadult males are transferred to other breeding centers or kept isolated [5].

The aim of the study was to compare immunoreactive fecal cortisol levels as they are an indicator of physiological stress levels in groups of captive European bison with varying social compositions and levels of visitor pressure. Knowledge on the social relations of the European bison was applied and the research was intentionally conducted in captive herds with a herd structure that was potentially conflictive or non-conflictive. The presence within the herd of a subadult male (i.e., about 3 years old) that could challenge older males was assumed to be a source of social conflicts. The hypothesis was formulated that social relations in a group of animals kept in captivity could be affected by visitor pressure and that this phenomenon would occur most in groups in which social conflicts (disorders) arise. Fecal glucocorticoid metabolite analyses are increasingly used in a variety of studies to examine stress hormone secretion in domestic and wild vertebrates [32, 33]. Moreover, when studying the impact of visitors on captive animals, this method is simple and promising, especially since the results of traditional behavioral observation studies may be less unequivocal [34].

The second aim was to determine if there is a correlation between intestinal parasitic burden and immunoreactive fecal cortisol levels. European bison are regularly dewormed at breeding centers, but their levels of parasitism can fluctuate greatly and even exceed the levels found in wild herds [35]. Although current research indicates a possible relation between parasitological infestation and the stress level of animals [36, 37], such studies have not yet been carried out on the European bison.

Over the last 100 years, the parasitic fauna of European bison has been thoroughly examined, and it is known that the most common parasites are nematodes from the Trichostrongylidae family, e.g., *Ostertagia ostertagi*, *Ostertagia leptospicularis*, *Ostertagia kolchida*, nematodes from the *Trichuris* genus, and coccidia from the *Eimeria* genus [38, 39].

## Methods

### Animals and study sites

When selecting the study sites, the following criteria were taken into account: (1) the studied herds of European bison should be family-like groups consisting of an adult male, adult females, and young individuals of both sexes; (2) groups should be characterized by varied social structures (based on interviews with animal keepers, captive herds with potentially conflictive or non-conflictive social structures were selected); (3) study sites should be characterized by cyclically variable visitor pressure (a greater number of visitors at weekends compared to weekdays); and (4) the enclosure layout and herd structure should ensure the possibility of taking fecal samples from identified individuals.

To simplify counting the number of visitors, daily ticket sales were analyzed. While this does not indicate whether a person actually visited the European bison enclosure, the number of tickets sold objectively indicates the trend in the number of visitors on weekdays and at weekends, and such trends are enough to reflect the level of visitors. After considering these factors and obtaining permits from administrators, the following locations were selected for the study: the zoos in Poznań and Warsaw, and the Forest Culture Center (FCC) in Gołuchów.

In these locations, animals were kept in typical group structures with one adult male, a few adult females, and young animals of both sexes (Table 1). However, in Warsaw Zoo and FCC Gołuchów, the herds also included a subadult male aged about 3 years. The three locations differed in terms of enclosure size and visitor numbers, and as a consequence the animal density per ha varied (Table 1). In FCC Gołuchów, European bison were allowed to use a 20 ha enclosure, but it was separated into two main pens: a daily pen of about 1 ha, where the animals were exposed to visitors, and a nighttime pen that was open to the animals at night. Visitor numbers significantly differed between working days and weekends: at weekends, there were 5–7 times more visitors (Table 1). On weekdays, there were no events that could affect the animals’ stress levels or unintentionally increase the number of visitors. The diet of the European bison differed between the locations (Table 2): cereals or a commercial fodder mixture were the basis of European bison's diet. At FCC Gołuchów and Warsaw Zoo, the fodder was more diverse and was supplemented with natural plant food. In all locations, hay or haylage was offered ad libitum.

**Table 1:** Composition of the European bison breeding group and enclosure conditions in each location (*during daytime, a pen of about 1 ha is available for the animals)

Location	Area of enclosure (ha)	Females (years old)	Males (years old)	Animals per ha	Mean Visitor numbers (persons/day)	
					Weekdays	Weekends
Poznań zoo	0.5	10, 8, 1.5	12, 1.5	10	19.5	99
Warsaw zoo	0.3	9, 9, 3, 2, 1, 0.5	11, 3	26.7	196.3	1087.5
FCC Gołuchów	20*	11, 11, 2	11, 3, 1, 1	0.35	90	650

**Table 2:** Composition of European bison diet in individual locations in winter and early spring (kg per adult individual)

Fodder	Poznań zoo	Warsaw zoo	FCC Gołuchów
Cereals	0.5	0.4	4.5
Commercial fodder mixture for livestock or European bison	2	0.8	–
Root crops	–	1.5	7.5
Seeds of trees	–	0.2	1
Leaves/branches	–	0.5	addition
Hay	ad libitum	ad libitum	4.5
Haylage	–	–	ad libitum

**Fig. 1:**
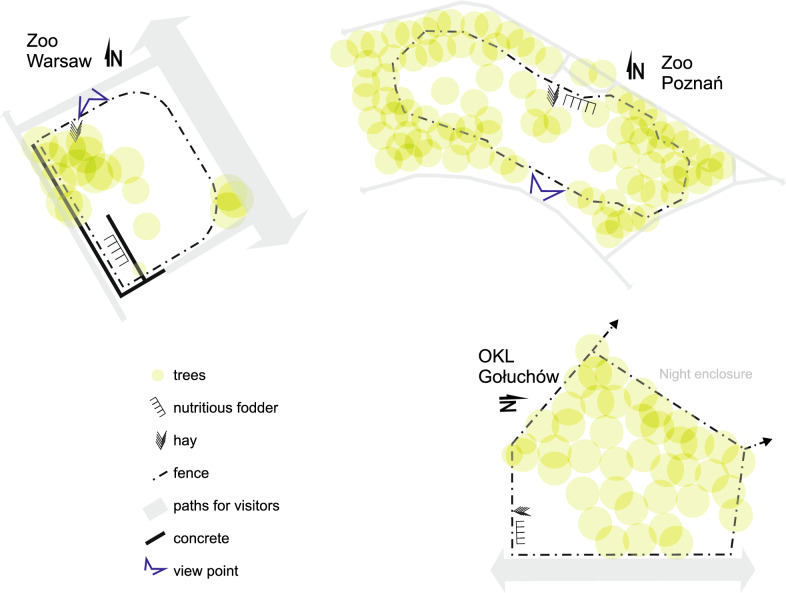
Maps of European bison enclosures in the three study sites. For FCC Gołuchów, only the daytime enclosure is presented

**Fig. 2:**
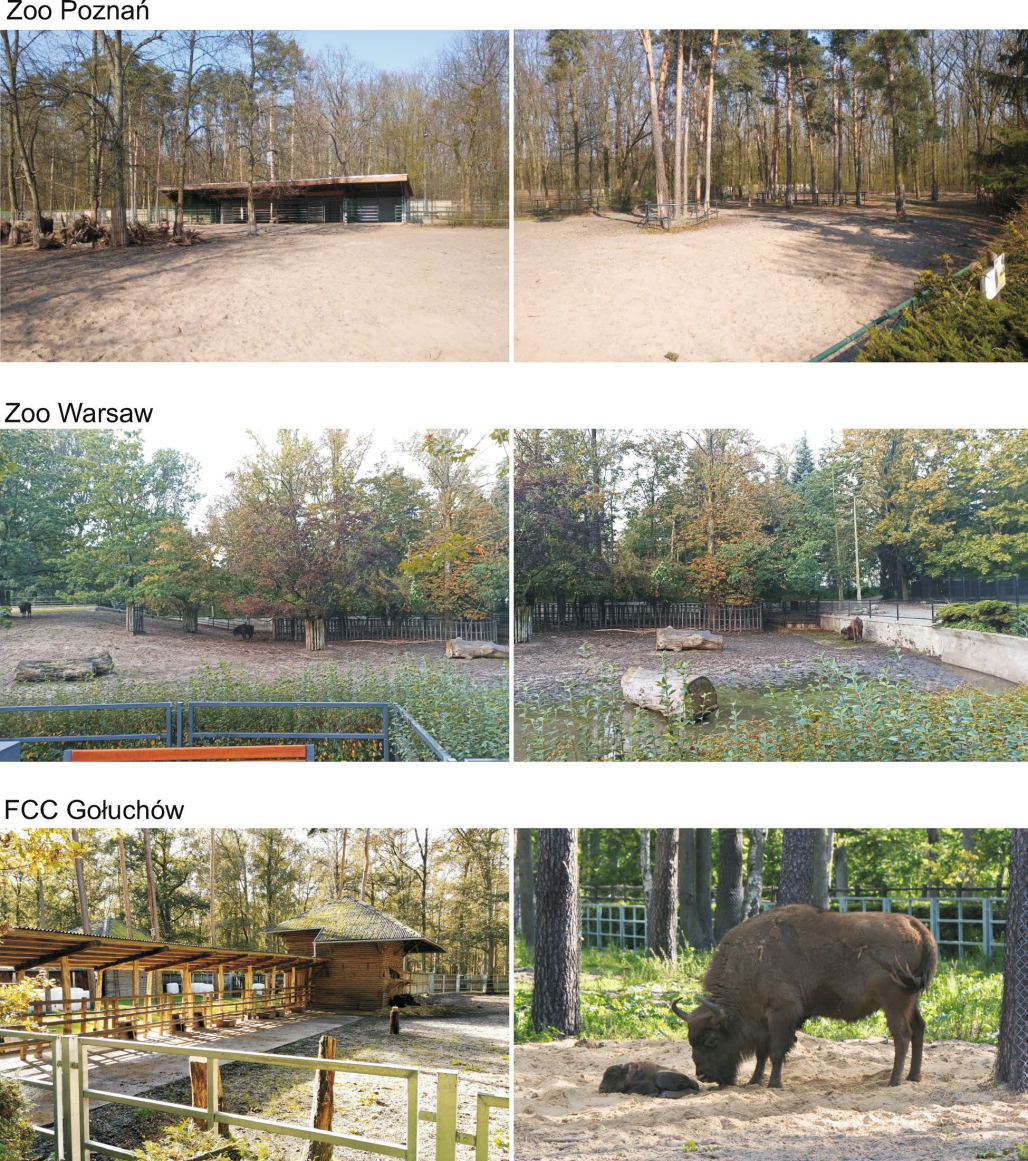
Photographic presentation of European bison enclosures in the three study sites

The enclosures were arranged in various ways (Fig. 1). In Warsaw Zoo, the enclosure was accessible to visitors from three sides, but fodder was given away from the view of visitors behind a concrete wall and an artificial hill. However, visitors could view animals feeding on hay near a viewpoint. In Poznań Zoo, the enclosure was surrounded by trees, but a viewpoint allowed good observation of the whole enclosure. Fodder was served in front of the viewpoint. At FCC Gołuchów, visitors had access to the enclosure from one side. The area was covered with trees, but these did not affect the visitors’ view. Feeders for these bison were located right next to the visitors’ path (Fig. 2). Visitors at Warsaw Zoo could observe animals from a height of about 2.5 m; at FCC Gołuchów this was about 1 m, but at Poznań Zoo visitors and bison were at the same height. In both zoos, animals were given nutritious fodder only once a day at about 8 am, before visitor facilities were open. At FCC Gołuchów, animals were given fodder twice a day: at 8 am and at about 4–5 pm).

### Study design

Samples were collected during the mild winter (December to February) in 2019–2020. Collection during this period allowed higher ambient temperatures and the reproductive period to be avoided, both of which could affect the level of immunoreactive fecal cortisol in animals [40–42]. The ambient temperature during material collection in the study ranged between 4 and 12 °C.

Fecal samples were collected for 5 consecutive days from Friday to Tuesday, except for the zoo in Poznań, where collection took place over 4 days (Saturday–Tuesday). Each day, samples were collected in the morning by animal keepers. Based on direct observation of the animals, each sample was assigned to the relevant individual, whose age and sex were known. Samples (2 mL, taken from the central part of the bolus) were frozen at − 20 °C, transported to the laboratory, and stored at the same temperature until the immunoreactive fecal cortisol level analysis. An additional 50 mL of feces was collected at Warsaw Zoo to assess parasitological infestation. These samples were cooled to 6 °C and stored until analysis. In total, 80 samples were collected from 19 animals for immunoreactive fecal cortisol analysis, and 33 samples were collected from 7 animals for assessment of parasitological infestation.

The level of immunoreactive fecal cortisol in the samples was measured using a non-specific commercial ELISA kit (COR ELISA Kit No. EU0391, competitive variant, Wuhan Fine Biological Technology Co., China). All details concerning the methodology of determining immunoreactive fecal cortisol have been described previously [43]. The analysis was performed using a Synergy 2 microplate reader* (BioTek Instruments, Inc. Winooski, Vermont, USA) equipped with an automated microplate strip washer* (ELx50, BioTek Instruments, Inc.) and an ELMI DTS-4 4-plate microplate thermo shaker* (ELMI SIA, Riga, Lithuania). All 80 samples were located within the range of the standard curve. Intra- and inter-assay coefficients of variation were 10.6% and 12.8%, respectively. Immunoreactive fecal cortisol levels were expressed as nanograms per one gram of dry mass of feces (ng/g). 1 g of fresh feces from each sample was weighed (with precision 0.001 g), dried at 60 °C for 24 h, and then weighed again (with the same precision) to assess the water content in each sample.

The commercial ELISA kit was biologically validated for the European bison using samples from three males that were transported from Poland to Spain. Two of the animals, named Plener III and Platyn, were taken from Pszczyna Park enclosure; one, named Podlas, was taken from FCC Gołuchów. The animals were transported in November to Encinarejo in Spain. The animals were 2–4 years old. In Pszczyna Park enclosure, no herd manipulations were carried out prior to transport. In FCC Gołuchów, Podlas was separated from the herd before transportation due to male aggression in the main enclosure. Before and after transportation, fecal samples were collected from all three animals. As the collection of fresh samples from European bison is difficult, sampling was carried out over several weeks. Finally, eight samples were collected from each individual and analyzed. We assumed that pre-transport sampling would provide information on the levels of immunoreactive fecal cortisol in the animals in their original environment. After transportation, however, efforts were made to collect samples in the first few days after the animals arrived. Samples were frozen at − 20 °C immediately after collection; they were then transported to the laboratory and stored at the same temperature until analysis. The experiment required the transport of samples from Spain to Poland in a transportable freezer at − 20 °C. The immunoreactive fecal levels were compared before and after the transport.

**Fig. 3:**
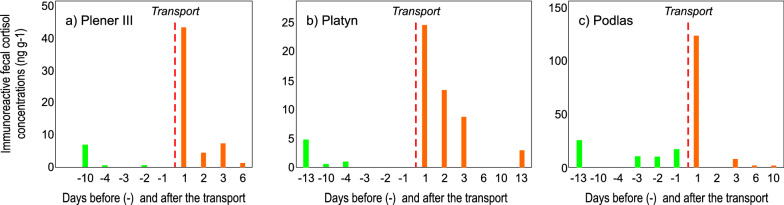
Immunoreactive fecal cortisol levels for samples collected from the three European bison bulls on the days before (marked as “-”) and after transport: **a** Plener III, **b** Platyn and **c** Podlas

All animals showed an increase in immunoreactive fecal cortisol level in fecal samples after transportation. Compared to the mean values before the transport, the level increased from 8 to 33 times on the first day after transport (Fig. 3). Compared to the pre-transport period, on the next 2 days (day 2 and 3) mean immunoreactive fecal cortisol levels in the fecal samples was 4.6 and 7 times higher for Plener III and Platyn, respectively (Fig. 3a, b). For Podlas, this value could not be determined because the material was not collected on the second day (Fig. 3c); this animal was separated from the herd before transportation and had already displayed increased immunoreactive fecal cortisol level in the pre-transport period. We were not able to statistically compare the immunoreactive fecal cortisol levels in the pre-transport and post-transport periods (n = 8 for each animal) because of the small sample size.

Three grams from each sample were examined for the presence of the eggs of gastrointestinal nematodes, and oocysts of coccidia from the genus *Eimeria* using McMaster’s quantitative method [44] in sucrose solution (SG = 1.27) [45]. The McMaster method can detect as few as 50 helminth eggs per gram of feces (EPG) and 50 *Eimeria* oocysts per gram of feces (OPG). Direct flotation was performed to identify the oocysts; their length and width were measured, and their morphology was analyzed. Descriptions of oocyst morphology and species differentiation followed the guidelines of Duszynski and Wilber [46] and Pyziel et al. [35, 38]. Samples were investigated using an OPTA-TECH Lab 40 light microscope at ×100 magnification for the McMaster method and ×100–400 magnification for direct flotation.

### Data processing and statistics

Sampling days were divided into two groups according to visitor pressure: low visitor pressure on weekdays and elevated pressure at weekends. When analyzing the data, it was considered that glucocorticoids, which are secreted into the blood, are excreted via the feces (in the form of fecal cortisol metabolites) after a delay. For livestock, this delay is about 10 to 24 h [33, 47, 48]. For this reason, because the samples were collected on consecutive days, the immunoreactive fecal cortisol level detected in a given sample represented the animal’s stress level on the previous day.

To assess the relation between immunoreactive fecal cortisol and parasitic burden, two dominant groups were used: *Eimeria* sp. and *Trichuris* sp*.* The relation was analyzed in two ways: directly (eggs or oocysts from the same fecal sample as the immunoreactive fecal cortisol level) and shifted days (comparison of eggs or oocysts against the fecal sample from the same animal on the next day). The direct relation (fecal samples from the same day) indicated the impact of the level of stress on egg/oocyst shedding (because glucocorticoids are evident in feces after a delay and thus represent the stress level for the previous day); the shifted comparison (eggs or oocysts counted in feces on the next day) indicated the impact of egg/oocyst shedding on immunoreactive fecal cortisol level.

Statistical analyses were performed in SPSS software. To reach normality, the response variable was Box-Cox transformed. The analysis of visitor impact at the selected locations was carried out with a general linear mixed model in which the immunoreactive fecal cortisol level was analyzed (as a dependent variable) with regard to four explanatory variables: (a) location (Poznań, Warsaw or Gołuchów); (b) visitor pressure (low and elevated number of visitors); (c) age of the animal; and (d) sex of the animal. We also added interactions between visitor pressure and all other variables: location, age and sex. In the model, the levels (weekdays and weekends) of visitors were used instead of the actual number of visitors. Because the conditions in the enclosures were not comparable, a similar number of visitors could impact these enclosures differently. Animal ID number was fitted as a random effect to account for the repeated sampling of individual animals. Restricted maximum likelihood (REML) was used to estimate the parameters in the best model obtained. Model selection was based on Akaike information criterion (AIC) values in a multi-model selection procedure [49]. All possible model permutations with all the explanatory variables were performed; finally, the models were ranked according to their Akaike weights (Additional file 1: Table S1). The principle of model selection was lower AIC values, but when the difference between the models was less than AIC = 2, the simpler model was chosen. A pairwise comparison with least significant difference (LSD) of groups was used in the factors that were statistically significant in the model. Analysis of the relation between parasitological infestation and immunoreactive fecal cortisol level was performed with Pearson's correlation coefficient (r, n = 33 for direct comparison and n = 25 for shifted comparison).

## Results

**Fig. 4:**
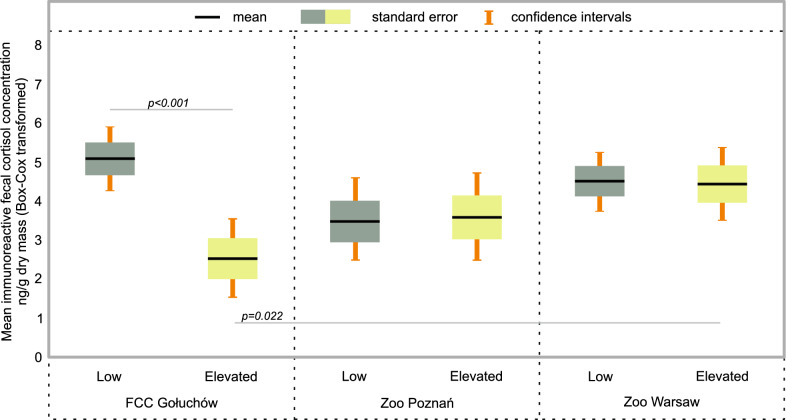
Effect of visitor pressure (Low = weekdays; Elevated = weekends) on immunoreactive fecal cortisol level (mean and 95% CI, Box–Cox transformed) in European bison with regard to site: low pressure—low numbers of visitors on weekdays; elevated pressure—5–7 times more visitors at weekends. Statistically significant differences in a pairwise comparison with the Least Difference Test are indicated on the graph

Immunoreactive fecal cortisol levels were not influenced by sex, age, or visitor pressure (P > 0.05; Table 3). However, study site and the interaction between study site and visitor pressure were statistically significant (P < 0.05). European bison in Gołuchów presented higher levels of immunoreactive fecal cortisol on weekdays than at weekends (P < 0.001; Fig. 4). Immunoreactive fecal cortisol levels of European bison housed in Poznań Zoo and Warsaw Zoo did not differ between weekdays and weekends (P = 0.972 and P = 0.943, respectively). At weekends, levels were higher in Warsaw Zoo than in Gołuchów (P = 0.022).

**Table 3:** Effect of site and visitors on immunoreactive fecal cortisol level in European bison in a general linear mixed model

Source	B	SE	t	P
Intercept	4.16	0.54	7.73	< 0.001*
Sex (F)	0.42	0.39	1.06	0.292
Sex (M)	0			
Site (FCC Gołuchów)	− 1.89	0.69	− 2.75	0.007
Site (Poznań zoo)	− 0.84	0.73	− 1.16	0.249
Site (Warsaw zoo)	0			
Visitors (low)	0.04	0.60	0.72	0.943
Visitors (elevated)	0			
Site*visitors (Gołuchów, Low)	2.59	0.88	2.93	0.005*
Site*visitors (Poznań, Low)	− 0.07	0.97	0.07	0.943
Site*visitors (Warsaw, Low)	0			

Non-significant age was excluded in the selection procedure

*Statistically significant

Parasitological examination showed the presence of eggs of gastrointestinal nematodes from the Trichostrongylidae family, nematodes from the *Trichuris* genus, and oocysts of five species of coccidia from the *Eimeria* genus (Table 4). Most prevalent were eggs from the *Trichuris* genus and oocysts of *Eimeria bovis*, both at weekends and on weekdays. Eggs from the Trichostrongylidae family were observed only on weekdays. All five species of coccidia were observed together only at weekends, whereas on weekdays only three species were found. The prevalence of all parasites except *Trichuris* sp. was equal or higher at weekends than on weekdays (Table 4.). The level of parasitic infestation and vanamnesis did not indicate any clinical signs of disease. No correlations between parasitic burden and immunoreactive fecal cortisol level were found in either the direct or the shifted comparison with regard to *Eimeria *sp. (r = − 0.038, P = 0.835 for direct; r = − 0.021, P = 0.921 for shifted) and *Trichuris *sp. (r = − 0.175, P = 0.329 for direct; r = 0.148, P = 0.479 for shifted).

**Table 4:** Parasitic infestations of European bison at Warsaw Zoo during five days of fecal sampling

Parasite	Weekdays			Weekend		
	Prevalence (%)	Intensity (EPG/OPG)		Prevalence (%)	Intensity (EPG/OPG)	
		Range	Median		Range	Median
Trichostrongylidae	14.29	50	50	0	0	0
*Trichuris* sp.	31.75	200–2200	550	35.71	50–1850	300
*Eimeria* sp.	52	50–300	225	71	50–1770	175

*EPG* helminth eggs per gram of feces, *OPG* *Eimeria* oocysts per gram of feces

## Discussion

Breeding conditions and visitor pressure may affect immunoreactive fecal cortisol levels in captive European bison. Average immunoreactive fecal cortisol levels in the studied captive herds ranged from 7.45 to 17.23 ng/g dry fecal mass. Investigating immunoreactive fecal cortisol levels in a larger number of captive and wild European bison herds and identifying the main factors responsible for the increase in stress hormone levels could be a valuable contribution to the conservation efforts that are undertaken for this species. This is important because the results indicate that there may be significant differences in the welfare of captive European bison kept under different conditions.

The results show that the studied range of visitor pressure did not increase levels of immunoreactive fecal cortisol in these captive European bison. None of the examined bison herds displayed higher immunoreactive fecal cortisol level at weekends despite a fivefold–sevenfold increase in the number of visitors to their enclosures, thus indicating that visitor pressure (in the studied range) does not negatively affect the welfare of European bison. This result corresponds with other studies on captive ruminants (in zoo conditions), in which only a slight reaction to visitors was shown [24]. However, these results differ from those obtained for other some ungulates (*Gazella soemmerringii*, *Bos gaurus*, *Cervus nippon* and *Antelope cervicapra)*, for which a negative response to increasing visitor pressure was found [20–23]. These discrepancies show that the response of captive wild animals to visitors can be species dependent [16, 50]. Moreover, individual traits may also be of great importance in the welfare of captive animals [51] and may impact their reaction to visitors [15].

One should also remember to consider the season in which the study was conducted and the related limitations of the results. The study was conducted during a mild winter, and it is difficult to predict on this basis whether the same reaction would occur in a hot summer period with an exceptionally large number of visitors. The ambient temperature during material collection in the study ranged between 4 and 12 °C. In Poland, the ambient temperature can exceed 35 °C in summer, and it can reach − 30 °C in winter [52]. The European bison is sensitive to high environmental temperatures [6, 53], which itself can probably cause physiological stress. Therefore, it is not possible to predict whether stimuli such as increased visitor pressure would have a greater physiological effect at high ambient temperatures. The lack of negative impact that was shown in this study occurred in moderate weather conditions and with moderate pressure from visitors.

The positive impact of visitors in Gołuchów indicates the role of local breeding conditions on the welfare of captive animals. This is probably the effect of social relations in the herd and, in particular, aggression between competing males. Competition between males in Gołuchów (in this case, between an 11-year-old male and a 3-year-old male) probably increased the stress levels of the whole herd. The differences observed between Gołuchów and Warsaw (in both these locations, the herds have a potentially conflictive social structure) show that more subtle factors than just the sex and age of the individuals in the herd may play an important role. The stress level could have been an individual effect. This phenomenon is indicated as an important element that affects animals' responses to breeding conditions [51, 54–56]. The male in Gołuchów was considered aggressive when the herd structure had been disturbed, which may further increase conflict within the group; however, this does not explain why a positive reaction to visitors occurred only in Gołuchów and not in Warsaw, both of which are herds with a conflictive structure. The probable reason may be the feeding system and the arrangement of the enclosures in which the animals were kept. In Gołuchów, feeders with nutritious fodder and hay were located near visitors, and fodder was also offered in the afternoon when visitors were present. The increased pressure from visitors at weekends probably distracted the competing males in Gołuchów and reduced the frequency or intensity of their aggressive behavior. Aggression usually occurs around feeding areas, where the instinct to monopolize resources occurs [57–59]. In addition, the animal feeding system in Gołuchów meant that more intense competitive male behavior could take place not only during morning feeding but also in the afternoon. As cortisol metabolites are excreted via feces after a delay [38, 48, 49], the possibility of finding elevated levels of immunoreactive fecal cortisol metabolites in Gołuchów was higher. This is important information when planning further research because it shows how many factors should be considered in order to correctly assess the welfare of the studied animals. If these suppositions are right, this could be another example of the positive impact of visitors on captive animals [14, 15, 18, 25], but it could also indicate the mitigating role of visitors in the social relations of captive animals as well as the importance of enclosure design and feeding systems.

The relationships between a herd’s social structure and stress levels illustrate the “physiological cost” of maintaining such conflict-causing systems. In free-ranging populations, bulls can reach sexual maturity at 3 years of age, but generally this happens at four years [60]. At the age of three, young bulls begin to move between mixed groups (formed by adult and young cows), and from the age of four they form separate male ‘bachelor’ herds [61], thus indicating that the currently recommended [5] removal of subadult males from breeding herds is justified. Similar disruptive incidents caused by conflicts among social animals have been reported for other species kept in captivity [62]. In the case of the European bison, more research is needed to study this phenomenon more thoroughly in various social groups. Moreover, the arrangement of enclosures and even feeding systems may be important as in some situations these may allow the use of visitor pressure to mitigate potential conflicts in the herd. It should also be remembered that short-term stress cannot be treated as something definitely negative because increases in cortisol levels are a natural and necessary element that mobilizes the body to act in the event of disruptions in the environment [63]. However, long-term stress can have a negative impact on animals as it can disrupt many aspects of animal physiology, e.g., reproduction, immune response, or hormonal control of the metabolism [63–65]. Understanding and alleviating or eliminating the potential factors responsible for chronic stress could be very desirable in terms of protecting this species, both in breeding conditions and in the wild.

Parasitic infections trigger the host’s immune system, thus activating defensive mechanisms; they are also suspected to affect the release of glucocorticoids. However, the influence of parasites on the host’s glucocorticoid level is still unclear as there are results that suggest both positive and negative correlations, or no relationship at all [66, 67]. According to the latest findings, it is advised to correlate fecal glucocorticoid metabolite levels with other factors such as the host’s age, sex and reproductive status, but also parasite type, infestation severity, food availability or social stressors [67]. Moreover, in an experimental infection of reindeer with gastrointestinal nematodes and subsequent parasitological treatment, it turned out that both infected and treated reindeer had similar levels of fecal glucocorticoid metabolites [68]. The authors suggested that these results could be explained by the tolerance strategy used by the host to cope with infestation. In our study, we found no correlation between parasite infestation and glucocorticoid levels, but this could be due to the low level of the parasite infestation. A host organism with a low parasite burden may be able to tackle the infestation with one of three protective strategies: avoidance, resistance or tolerance [69].

Other limitations of the study should be also pointed out. The exact delay between cortisol circulation in the blood and excretion into feces is not known for the European bison. Some studies indicate a high inter-individual variability in such delays (e.g., [70]), which could also have impacted our results. Our study would have been strengthened by including behavioral observations and more precise visitor numbers.

## Conclusions

The study determined immunoreactive fecal cortisol levels in various captive herds of European bison. Moderate visitor pressure did not increase the stress levels of the studied animals. However, we found that the increased presence of visitors probably modified the social relations in a herd in a positive way. More subtle factors, such as individual animal characteristics, feeding systems, and the arrangement of enclosures, can be of great importance in terms of the effect of visitors on animals. The results can be used in guidelines for the management of European bison populations. Nevertheless, further research is needed to better understand the stress reactions of European bison in different seasons (thermal conditions), with different social structures of herds, with varying visitor pressure, and with behavioral observations. Additional investigation is also needed concerning the relation between parasite infestation and stress levels in European bison; however, this should be performed on animals whose infestation levels are more varied.

## Supplementary Information

**Additional file 1: Table S1.** Ranking of the models (including null model) explaining the immunoreactive fecal cortisol level in European bison (ΔAIC—AIC differences, ω_i_—Akaike weights, Rank—rank of the models based on AIC values, SITE—location (Poznań, Warsaw or Gołuchów); VISITORS—visitor pressure (low and elevated number of visitors), AGE—age of animals, SEX—sex of animals); bolded text in a row indicates the chosen model.

## Data Availability

The datasets used and/or analysed during the current study are available from the corresponding author on reasonable request.
