# Supplementary material for: Stress hormone level and the welfare of captive European bison (Bison bonasus): the effects of visitor pressure and the social structure of herds
Source: Acta Vet Scand. 2021 Jun 10;63:24. doi: 10.1186/s13028-021-00589-9 (PMC8193117; doi:10.1186/s13028-021-00589-9)
Supplement: Supplementary file 1 — Additional file 1: Table S1. Ranking of the models (including null model) explaining the immunoreactive fecal cortisol level in European bison (ΔAIC—AIC differences, ωi—Akaike weights, Rank—rank of the models based on AIC values, SITE—location (Poznań, Warsaw or Gołuchów); VISITORS—visitor pressure (low and elevated number of visitors), AGE—age of animals, SEX—sex of animals); bolded text in a row indicates the chosen model. [file 13028_2021_589_MOESM1_ESM.pdf]

Supplementary Table 2

Ranking of the models (including null model) within 95%confidence intervals ($\sum\omega_i$=0.95) explaining the immunoreactive fecal cortisol concentration in European bison (ΔAIC - AIC differences, ω_i - Akaike weights, Rank - rank of the models based on AIC values, SITE - location (Poznań, Warsaw or Gołuchów); VISIT - visitor pressure (low and elevated number of visitors), AGE – age of animals, SEX – sex of animals, * - interaction); bolded text in the row indicates chosen model.

Model	ΔAIC	ω_i	Rank
SITE + VISIT + SEX+ SITE*VISIT + SEX*VISIT	0.000	0.380	1
**SITE + VISIT + SEX+ SITE*VISIT**	**0.907**	**0.242**	**2**
SITE + VISIT + SITE*VISIT	2.010	0.139	3
SITE + VISIT + SEX + AGE + SITE*VISIT + SEX*VISIT	3.356	0.071	4
SITE + VISIT + SEX + AGE + SITE*VISIT + SEX*VISIT + AGE*VISIT	3.482	0.067	5
SITE + VISIT + SEX + AGE + SITE*VISIT + AGE*VISIT	4.446	0.041	6
SITE + VISIT + SEX + AGE + SITE*VISIT	5.269	0.027	7
null model	23.302	0.000	30
